# Risk for ischemic stroke and coronary heart disease associated with migraine and migraine medication among older adults

**DOI:** 10.1186/s10194-021-01338-z

**Published:** 2021-10-13

**Authors:** Emily C. McKinley, Christine L. Lay, Robert S. Rosenson, Ligong Chen, Victoria Chia, Lisandro D. Colantonio, Paul Muntner, Robert Urman, Michael E. Farkouh

**Affiliations:** 1 Department of Epidemiology, University of Alabama at Birmingham, 1665 University Blvd, RPHB 523B, Birmingham, AL 35233-0013 USA; 2 Centre for Headache, University of Toronto, Toronto, ON Canada; 3 Icahn School of Medicine at Mount Sinai, New York, NY USA; 4 Amgen Inc, Thousand Oaks, CA USA; 5 Peter Munk Cardiac Centre and Heart and Stroke Richard Lewar Centre, University of Toronto, Toronto, Canada

**Keywords:** Migraine, Myocardial infarction, Coronary revascularization, Ischemic stroke

## Abstract

**Background:**

Migraine has been associated with cardiovascular disease (CVD) events among middle-aged adults. The objective of this study was to determine the risk for ischemic stroke and coronary heart disease (CHD) events among older adults with versus without migraine.

**Methods:**

This retrospective cohort study was conducted using data from US adults ≥66 years of age with Medicare health insurance between 2008 and 2017. After stratification by history of CVD, patients with a history of migraine were matched 1:4 to those without a history of migraine, based on calendar year, age, and sex. Patients were followed through December 31, 2017 for ischemic stroke and CHD events including myocardial infarction or coronary revascularization. All analyses were done separately for patients with and without a history of CVD.

**Results:**

Among patients without a history of CVD (*n* = 109,950 including *n* = 21,990 with migraine and *n* = 87,960 without migraine), 1789 had an ischemic stroke and 3552 had a CHD event. The adjusted hazard ratio (HR) among patients with versus without migraine was 1.20 (95% confidence interval [95%CI], 1.07–1.35) for ischemic stroke and 1.02 (95%CI, 0.93–1.11) for CHD events. Compared to patients without migraine, those with migraine who were taking an opioid medication had a higher risk for ischemic stroke (adjusted HR 1.43 [95%CI, 1.20–1.69]), while those taking a triptan had a lower risk for CHD events (adjusted HR 0.79 [95%CI, 0.67–0.93]). Among patients with a history of CVD (*n* = 79,515 including *n* = 15,903 with migraine and *n* = 63,612 without migraine), 2960 had an ischemic stroke and 7981 had a CHD event. The adjusted HRs (95%CI) for ischemic stroke and CHD events associated with migraine were 1.27 (1.17–1.39) and 0.99 (0.93–1.05), respectively. Patients with migraine taking an opioid medication had a higher risk for ischemic stroke (adjusted HR 1.21 [95%CI, 1.07–1.36]), while those taking a triptan had a lower risk for CHD events (adjusted HR 0.83 [95%CI, 0.72–0.95]), each versus those without migraine.

**Conclusions:**

Older adults with migraine are at increased risk for ischemic stroke. The risk for ischemic stroke among older adults with migraine may differ by migraine medication classes.

**Supplementary Information:**

The online version contains supplementary material available at 10.1186/s10194-021-01338-z.

## Introduction

Studies of middle-aged adults have shown migraine to be associated with an increased risk for cardiovascular disease (CVD) events, including ischemic stroke and coronary heart disease (CHD) events [1]. The mechanisms linking migraine to CVD events are unclear, but have been speculated to include cortical spreading depression, hypercoagulation, endothelial dysfunction, shared genetic risk, vasospasm, or a higher prevalence of cardiovascular risk factors among patients with migraine [2, 3]. The risk for CVD events is higher among older versus middle-aged adults [4]. However, many risk factors for CVD events in middle-aged adults, including serum cholesterol and blood pressure, are not associated with increased CVD risk in older adults [5]. Little is known about the risk for CVD events associated with migraine in older adults [1, 6].

It has been suggested that certain classes of medications used to treat migraine, including triptans and ergotamines, are associated with an increased risk for CVD events [6, 7]. Also, specific migraine medications, including triptans, are contra-indicated for some patients with a history of CVD or high CVD risk [8, 9]. Few data are available on the risk for CVD events among older adults taking different classes of migraine medication [10]. A better understanding of the CVD risk associated with migraine and the use of migraine medications in older adults may advance knowledge of, and inform appropriate use of, migraine therapies in this population.

The primary objective of the current study was to compare the risk for ischemic stroke and CHD events among older US adults with versus without migraine. Since an association between some migraine medications and an increased risk for CVD events has been reported, we also analyzed the risk for ischemic stroke and CHD events among older adults with migraine taking different migraine medication classes [11–14]. As the risk for CVD events is higher among adults with versus without a history of CVD [15], all analyses were conducted for patients with and without a history of CVD, separately.

## Methods

We conducted a retrospective cohort study using data from a 5 % random sample of Medicare beneficiaries. Medicare is a government health insurance program for US adults aged 65 years and older, and adults younger than 65 years with end-stage renal disease or who are disabled. Medicare data were obtained from the Centers for Medicare and Medicaid Services (CMS) Chronic Condition Data Warehouse. The institutional review board at the University of Alabama at Birmingham approved the study and waived the requirement to obtain informed consent.

### Study population

We identified Medicare beneficiaries with a history of migraine between January 1, 2008 and December 31, 2017, using inpatient and outpatient healthcare utilization claims for migraine and pharmacy fills for acute migraine medications as defined in Supplemental Table 1. To provide adequate time to identify patient characteristics including the presence of comorbidities, we restricted the study population to Medicare beneficiaries who were living in the US and had continuous inpatient, outpatient, and pharmacy coverage for the 365 days prior to meeting the definition of history of migraine. As we were interested in studying history of migraine in older adults, we further restricted the study population to Medicare beneficiaries ≥66 years of age on the date they met the definition of history of migraine which means they were ≥ 65 years of age 365 days prior to meeting the definition of history of migraine (i.e., at the start of the look-back period). We required each beneficiary to be alive on the date they met the criteria for having a history of migraine. For each beneficiary, the earliest date they met all of the inclusion criteria described above was defined as their index date.

To serve as a control group, we identified beneficiaries who did not have a history of migraine, as defined above, using all available Medicare claims on or before December 31, 2017. For each beneficiary without a history of migraine, we randomly selected a day between January 1, 2008 and December 31, 2017 to serve as their index date. We applied identical inclusion/exclusion criteria for beneficiaries without a history of migraine as for those with a history of migraine. Specifically, we restricted the analysis to patients without a history of migraine who lived in the US and had continuous inpatient, outpatient, and pharmacy coverage for the 365 days before their index date. We further restricted the analysis to beneficiaries ≥66 years of age who were alive on their index date. For patients with and without a history of CVD, separately, beneficiaries without a history of migraine were frequency matched to those with a history of migraine four to one based on the calendar year of their index date, age in years on their index date, and sex.

### Patient characteristics

We used Medicare beneficiary and claims data between January 1, 2007 and each patient’s index date to define characteristics (Supplemental Table 2). In addition to age on their index date, sex, and history of CVD, patient characteristics analyzed included race/ethnicity, receiving a low-income subsidy to pay for their health insurance, smoking status, history of diabetes, hypertension, chronic kidney disease (CKD), heart failure, dementia, depression, an anxiety disorder, insomnia, cancer, epilepsy, and hospitalization within the past year. Among beneficiaries with migraine, we further identified those with a history of migraine with aura. Beneficiaries who did not meet the definition for comorbidities listed above were assumed not to have those conditions. Area-level income was defined by the median income level within the patient’s zip code of residence according to data from the 2017 American Community Survey [16]. We also used Medicare pharmacy claims within 90 days prior to each patient’s index date to identify the use of antihypertensive medication, glucose-lowering medication, statins, non-statin lipid-lowering medications, medications for insomnia, and, among women, hormone replacement therapy. A list of these medications is provided in Supplemental Table 3.

### Migraine medication use

For patients with a history of migraine, we used Medicare pharmacy claims within 90 days prior to each patient’s index date to identify use of acute migraine medications (i.e., triptans, ergotamine, nonsteroidal anti-inflammatory drugs [NSAID], and opioids) and preventive migraine medications (i.e., select antiepileptic agents, select antihypertensive agents, select antidepressants, botulinum toxin, and other agents). A list of the medications in each of these classes is provided in Supplemental Table 4.

### Cardiovascular outcomes

Patients were followed from their index date through December 31, 2017 for ischemic stroke events and CHD events (i.e., myocardial infarction hospitalization or coronary revascularization), separately, with censoring occurring if they lost Medicare fee-for-service inpatient or outpatient coverage or died. The definitions of ischemic stroke and CHD events are provided in Supplemental Table 5. As a secondary outcome, we analyzed the risk for CVD events, defined as the composite of an ischemic stroke or CHD event.

### Statistical analysis

All statistical analyses described below were conducted among Medicare beneficiaries with and without a history of CVD, separately. We calculated summary statistics of patient characteristics for those with and without a history of migraine. We calculated the cumulative incidence and incidence rates of ischemic stroke, CHD and CVD events for patients with and without a history of migraine accounting for the competing risk of all-cause mortality as described by Fine and Gray [17]. We used Cox proportional hazard models with the Breslow method for tie handling to estimate hazard ratios (HR) and 95% confidence intervals (CI) for the risk of ischemic stroke, CHD events, and composite CVD events among patients with versus without a history of migraine. Four models with progressive adjustment for covariates were used. Model 1 was unadjusted. Model 2 included adjustment for age, sex, and race/ethnicity. Model 3 included adjustment for age, sex, race/ethnicity, low-income subsidy, area-level income, smoking status, diabetes, hypertension, CKD, heart failure, dementia, depression, insomnia, cancer, epilepsy, and hospitalization within the past year. Model 4 included adjustment for the variables in the third model and use of antihypertensive medication, glucose-lowering medication, statins, non-statin lipid-lowering therapy, medication for insomnia, and among women, hormone replacement therapy. As prior studies have shown an association between migraine with aura and increased risk of ischemic stroke [18, 19], we also estimated incidence rates and HRs for ischemic stroke associated with a history of migraine with and without aura, separately.

The calculation of rates and HRs for ischemic stroke, CHD events, and CVD events described above was repeated comparing patients with migraine but not taking migraine medications, those with migraine taking each migraine medication class and taking two or more migraine medication classes, each versus their counterparts without migraine. Few patients with migraine with and without a history of CVD were using ergotamines or botulinum toxin and the number of outcome events during follow-up in these subgroups was low. Therefore, we did not analyze the risk for outcome events among patients with migraine taking ergotamines or botulinum toxin. All analyses were conducted using SAS 9.4 (SAS Institute) with a two-sided level of statistical significance of 0.05.

## Results

**Fig. 1:**
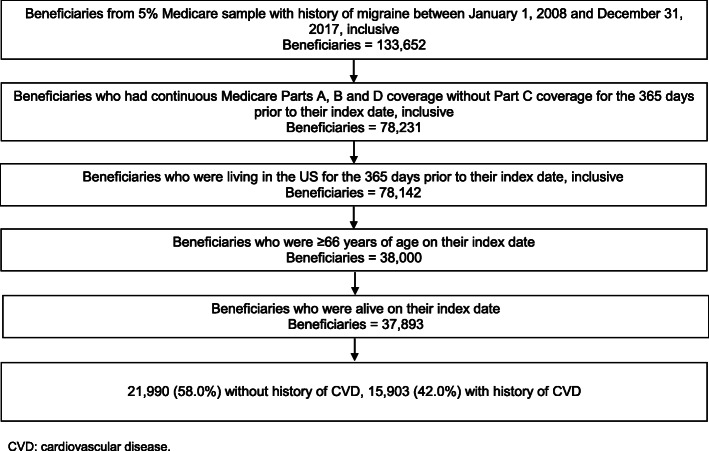
Consort diagram of Medicare beneficiaries with a history of migraine included and not included in the current analysis. CVD: cardiovascular disease

Overall, 37,893 Medicare beneficiaries with a history of migraine met the inclusion criteria for the current study, including 21,990 without a history of CVD and 15,903 with a history of CVD (Fig. 1). The 21,990 patients with a history of migraine who did not have a history of CVD were matched to 87,960 patients without a history of migraine or CVD. Also, the 15,903 patients with a history of migraine who had a history of CVD were matched to 63,612 patients without a history of migraine with a history of CVD.

### Patients without a history of CVD

Compared to their counterparts without a history of migraine, patients with a history of migraine were more likely to have depression, an anxiety disorder, and insomnia, and to have been hospitalized in the prior year (Table 1). Patients with a history of migraine were also more likely to take medications for insomnia and, among women, hormone replacement therapy.

**Table 1:** Characteristics of patients without a history of cardiovascular disease who had and did not have a history of migraine

	History of Migraine	
	No (*n* = 87,960)	Yes (*n* = 21,990)
Calendar year of the index date, N (%)		
2008	9268 (10.5)	2317 (10.5)
2009	5648 (6.4)	1412 (6.4)
2010	5368 (6.1)	1342 (6.1)
2011	6076 (6.9)	1519 (6.9)
2012	6596 (7.5)	1649 (7.5)
2013	8200 (9.3)	2050 (9.3)
2014	10,744 (12.2)	2686 (12.2)
2015	10,348 (11.8)	2587 (11.8)
2016	12,164 (13.8)	3041 (13.8)
2017	13,548 (15.4)	3387 (15.4)
Age, years, N (%)		
66–70	55,096 (62.6)	13,774 (62.6)
71–75	16,948 (19.3)	4237 (19.3)
76–80	8660 (9.8)	2165 (9.8)
81–85	4504 (5.1)	1126 (5.1)
≥ 86	2752 (3.1)	688 (3.1)
Age, years, mean (SD)	71.3 (5.6)	70.8 (5.8)
Females, N (%)	74,616 (84.8)	18,654 (84.8)
Race/Ethnicity, N (%)		
Non-Hispanic Black	6353 (7.2)	818 (3.7)
Non-Hispanic White	75,573 (85.9)	20,030 (91.1)
Hispanic	1435 (1.6)	317 (1.4)
Asian	1750 (2.0)	211 (1.0)
Other	2849 (3.2)	614 (2.8)
Low-income subsidy, N (%)	19,343 (22.0)	4135 (18.8)
Area level median income, N (%)		
< $35,000	6204 (7.1)	1193 (5.4)
$35,000 - $49,999	26,285 (29.9)	5935 (27.0)
$50,000 - $74,999	33,563 (38.2)	8476 (38.5)
≥ $75,000	21,908 (24.9)	6386 (29.0)
Smoking, N (%)	6783 (7.7)	2669 (12.1)
Diabetes, N (%)	19,873 (22.6)	3780 (17.2)
Hypertension, N (%)	55,190 (62.7)	13,264 (60.3)
Chronic kidney disease, N (%)	11,009 (12.5)	3132 (14.2)
History of heart failure, N (%)	3240 (3.7)	825 (3.8)
Depression, N (%)	8783 (10.0)	4730 (21.5)
Anxiety disorders, N (%)	8008 (9.1)	4222 (19.2)
Insomnia, N (%)	3865 (4.4)	2141 (9.7)
History of dementia, N (%)	3776 (4.3)	999 (4.5)
Cancer, N (%)	13,191 (15.0)	4231 (19.2)
Epilepsy, N (%)	1669 (1.9)	993 (4.5)
Hospitalization in the prior year, N (%)	8714 (9.9)	5323 (24.2)
Medication use, N (%)		
Antihypertensive medication	48,837 (55.5)	11,887 (54.1)
Glucose-lowering medication	11,987 (13.6)	2063 (9.4)
Statins	29,473 (33.5)	7247 (33.0)
Non-statin lipid-lowering medication	2901 (3.3)	793 (3.6)
Medication for insomnia	11,604 (13.2)	7039 (32.0)
Hormone replacement therapy among women	4473 (6.0)	2260 (12.1)
Migraine with aura, N (%)	–	2657 (12.1)

*SD* standard deviation

**Fig. 2:**
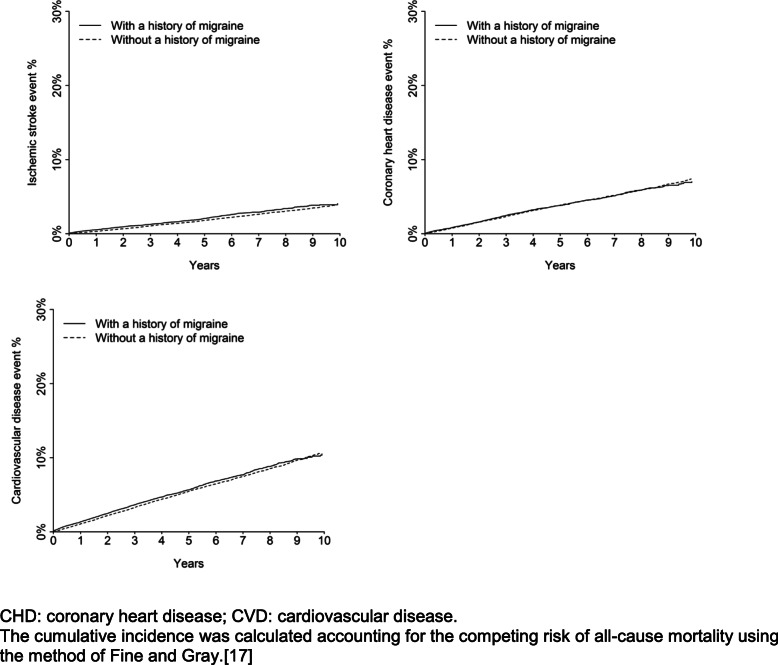
Cumulative incidence ischemic stroke, coronary heart disease and cardiovascular events among patients with and without a history of migraine, restricted to those without a history of cardiovascular disease. CHD: coronary heart disease; CVD: cardiovascular disease. The cumulative incidence was calculated accounting for the competing risk of all-cause mortality using the method of Fine and Gray [17].

Over a median follow-up time of 3.4 years (maximum of 10 years), among patients with and without a history of migraine, there were 406 and 1383 ischemic stroke events, respectively, 720 and 2832 CHD events, respectively, and 1084 and 4050 CVD events, respectively. The cumulative incidence of ischemic stroke was higher among patients with versus without a history of migraine (Fig. 2). There was no evidence of a difference in the cumulative incidence of CHD or CVD between patients with versus without a history of migraine. After multivariable adjustment, history of migraine was associated with a higher risk for ischemic stroke (HR 1.20 [95% CI, 1.07–1.35]) and CVD events (HR 1.08 [95% CI, 1.01–1.16]) but not CHD events (HR 1.02 [95% CI, 0.93–1.11]) (Table 2). Compared to those without a history of migraine, the multivariable-adjusted HR for ischemic stroke events was 1.21 (95% CI, 1.07–1.37) for patients with a history of migraine without aura and 1.18 (95% CI, 0.88–1.57) for patients with a history of migraine with aura (Supplemental Table 6).

**Table 2:** Incidence rates and hazard ratios for ischemic stroke, coronary heart disease and cardiovascular disease events associated with a history of migraine among patients without a history of cardiovascular disease

	History of migraine	
	No (*n* = 87,960)	Yes (*n* = 21,990)
Ischemic stroke		
Number of events	1383	406
Follow-up in person-years	342,211	86,726
Incidence rate (95% CI)^a^	4.0 (3.8, 4.3)	4.7 (4.2, 5.1)
Hazard ratio (95% CI)		
Model 1	1 (ref)	1.16 (1.03, 1.29)
Model 2	1 (ref)	1.21 (1.08, 1.35)
Model 3	1 (ref)	1.21 (1.08, 1.36)
Model 4	1 (ref)	1.20 (1.07, 1.35)
Coronary heart disease^b^		
Number of events	2832	720
Follow-up in person-years	338,452	85,866
Incidence rate (95% CI)^a^	8.4 (8.1, 8.7)	8.4 (7.8, 9.0)
Hazard ratio (95% CI)		
Model 1	1 (ref)	1.00 (0.92, 1.09)
Model 2	1 (ref)	1.01 (0.93, 1.09)
Model 3	1 (ref)	1.01 (0.93, 1.10)
Model 4	1 (ref)	1.02 (0.93, 1.11)
Composite CVD events		
Number of events	4050	1084
Follow-up in person-years	335,734	84,942
Incidence rate (95% CI)^a^	12.1 (11.7, 12.4)	12.8 (12.0, 13.5)
Hazard ratio (95% CI)		
Model 1	1 (ref)	1.06 (0.99, 1.13)
Model 2	1 (ref)	1.08 (1.01, 1.15)
Model 3	1 (ref)	1.08 (1.01, 1.16)
Model 4	1 (ref)	1.08 (1.01, 1.16)

^a^Rates are expressed per 1000 person-years

^b^Includes myocardial infarction or coronary revascularization

Model 1: Unadjusted model

Model 2: Adjusted for age, sex, and race/ethnicity

Model 3: Adjusted for variables in the second model and low income, area-level income, smoking, diabetes, hypertension, CKD, history of heart failure, dementia, depression, insomnia, cancer, epilepsy, and hospitalization within the past year

Model 4: Adjusted for variables in Model 3 and use of antihypertensive medication, diabetes medication, barbiturates, benzodiazepines, antihistamine medication for insomnia, non-benzodiazepine medication for insomnia, sedative hypnotics, and sedative antidepressants, statins, non-statin lipid-lowering therapy, and hormone replacement therapy

Among patients with a history of migraine, opioids and triptans were the acute migraine medications most commonly used, being taken by 36% of patients for both therapies, while antihypertensive medications were the most commonly used migraine-preventive therapy (Supplemental Table 7). After multivariable adjustment, patients with a history of migraine taking an opioid medication had a higher risk for ischemic stroke and CHD events versus their counterparts without a history of migraine. Patients with a history of migraine taking a triptan had a lower risk for CHD events versus those without a history of migraine, but there was no evidence of a difference in the risk for ischemic stroke.

### Patients with a history of CVD

Among patients with a history of CVD, those who had a history of migraine were more likely to have depression, an anxiety disorder, and insomnia, and to have been hospitalized in the prior year compared to their counterparts without a history of migraine (Table 3). Patients with a history of migraine were also more likely to be taking medication for insomnia.

**Table 3:** Characteristics of patients with a history of cardiovascular disease who had and did not have a history of migraine

	History of Migraine	
	No (*n* = 63,612)	Yes (*n* = 15,903)
Calendar year of the index date, N (%)		
2008	6860 (10.8)	1715 (10.8)
2009	4560 (7.2)	1140 (7.2)
2010	4668 (7.3)	1167 (7.3)
2011	5620 (8.8)	1405 (8.8)
2012	6032 (9.5)	1508 (9.5)
2013	5972 (9.4)	1493 (9.4)
2014	7276 (11.4)	1819 (11.4)
2015	6652 (10.5)	1663 (10.5)
2016	7708 (12.1)	1927 (12.1)
2017	8264 (13.0)	2066 (13.0)
Age, years, N (%)		
66–70	21,352 (33.6)	5338 (33.6)
71–75	15,204 (23.9)	3801 (23.9)
76–80	12,096 (19.0)	3024 (19.0)
81–85	8456 (13.3)	2114 (13.3)
≥ 86	6504 (10.2)	1626 (10.2)
Age, years, mean (SD)	75.4 (7.3)	75.1 (7.2)
Females, N (%)	48,904 (76.9)	12,226 (76.9)
Race/Ethnicity, N (%)		
Non-Hispanic Black	6277 (9.9)	1105 (6.9)
Non-Hispanic White	53,070 (83.4)	13,833 (87.0)
Hispanic	1434 (2.3)	395 (2.5)
Asian	1404 (2.2)	259 (1.6)
Other	1427 (2.2)	311 (2.0)
Low-income subsidy, N (%)	23,437 (36.8)	6075 (38.2)
Area level median income, N (%)		
< $35,000	6396 (10.1)	1508 (9.5)
$35,000 - $49,999	21,397 (33.6)	5240 (32.9)
$50,000 - $74,999	23,010 (36.2)	5659 (35.6)
≥ $75,000	12,809 (20.1)	3496 (22.0)
Smoking, N (%)	9446 (14.8)	3648 (22.9)
Diabetes, N (%)	28,424 (44.7)	6455 (40.6)
Hypertension, N (%)	58,817 (92.5)	14,740 (92.7)
Chronic kidney disease, N (%)	22,642 (35.6)	6180 (38.9)
History of heart failure, N (%)	19,115 (30.0)	4871 (30.6)
Depression, N (%)	10,197 (16.0)	4633 (29.1)
Anxiety disorders, N (%)	8682 (13.6)	4273 (26.9)
Insomnia, N (%)	3832 (6.0)	1823 (11.5)
History of dementia, N (%)	9243 (14.5)	2519 (15.8)
Cancer, N (%)	11,064 (17.4)	3429 (21.6)
Epilepsy, N (%)	3547 (5.6)	1896 (11.9)
Hospitalization in the prior year, N (%)	18,033 (28.3)	8119 (51.1)
Medication use, N (%)		
Antihypertensive medication	51,432 (80.9)	12,587 (79.1)
Glucose-lowering medication	15,089 (23.7)	3009 (18.9)
Statins	34,512 (54.3)	8230 (51.8)
Non-statin lipid-lowering medication	3830 (6.0)	987 (6.2)
Medication for insomnia	12,014 (18.9)	5576 (35.1)
Hormone replacement therapy among women	1918 (3.0)	834 (6.8)
Migraine with aura, N (%)	–	2245 (14.1)

*SD* standard deviation

**Fig. 3:**
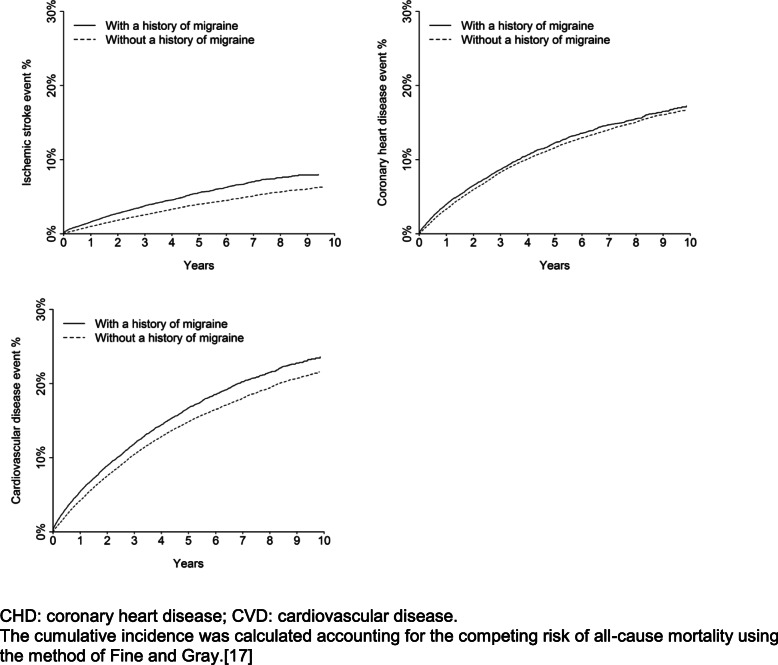
Cumulative incidence of ischemic stroke, coronary heart disease and cardiovascular events among patients who had and did not have a history of migraine, restricted to those with a history of cardiovascular disease. CHD: coronary heart disease; CVD: cardiovascular disease. The cumulative incidence was calculated accounting for the competing risk of all-cause mortality using the method of Fine and Gray [17].

Over a median follow-up time of 3.1 years (maximum of 10 years), among patients with and without a history of migraine, there were 765 and 2195 ischemic stroke events, respectively, 1677 and 6304 CHD events, respectively and 2301 and 8062 CVD events, respectively. The cumulative incidence of ischemic stroke and CVD events but not CHD events was higher among patients with a history of migraine versus without a history of migraine (Fig. 3). After multivariable adjustment, a history of migraine was associated with a higher risk for ischemic stroke (HR 1.27 [95% CI, 1.17–1.39]) and CVD events (HR 1.06 [95% CI, 1.01–1.12]) but not for CHD events (HR 0.99 [95% CI, 0.93–1.05]) (Table 4). After multivariable adjustment and compared to patients without a history of migraine, the HR for ischemic stroke events was 1.27 (95% CI, 1.16–1.39) and 1.29 (95% CI, 1.06–1.57) for patients with a history of migraine without and with aura, respectively (Supplemental Table 8).

**Table 4:** Incidence rates and hazard ratios for ischemic stroke, coronary heart disease and cardiovascular disease events associated with a history of migraine among patients with a history of cardiovascular disease

	History of migraine	
	No (*n* = 63,612)	Yes (n = 15,903)
Ischemic stroke		
Number of events	2195	765
Follow-up in person-years	227,257	58,097
Incidence rate (95% CI)^a^	9.7 (9.3, 10.1)	13.2 (12.2, 14.1)
Hazard ratio (95% CI)		
Model 1	1 (ref)	1.37 (1.26, 1.48)
Model 2	1 (ref)	1.39 (1.28, 1.51)
Model 3	1 (ref)	1.25 (1.15, 1.36)
Model 4	1 (ref)	1.27 (1.17, 1.39)
Coronary heart disease^b^		
Number of events	6304	1677
Follow-up in person-years	217,059	55,859
Incidence rate (95% CI)^a^	29.0 (28.3, 29.8)	30.0 (28.6, 31.5)
Hazard ratio (95% CI)		
Model 1	1 (ref)	1.04 (0.99, 1.10)
Model 2	1 (ref)	1.04 (0.98, 1.09)
Model 3	1 (ref)	0.99 (0.93, 1.04)
Model 4	1 (ref)	0.99 (0.93, 1.05)
Composite CVD events		
Number of events	8062	2301
Follow-up in person-years	213,557	54,347
Incidence rate (95% CI)^a^	37.8 (36.9, 38.6)	42.3 (40.6, 44.1)
Hazard ratio (95% CI)		
Model 1	1 (ref)	1.13 (1.08, 1.18)
Model 2	1 (ref)	1.13 (1.08, 1.18)
Model 3	1 (ref)	1.06 (1.01, 1.11)
Model 4	1 (ref)	1.06 (1.01, 1.12)

^a^Rates are expressed per 1000 person-years

^b^Includes myocardial infarction or coronary revascularization

Model 1: Unadjusted model

Model 2: Adjusted for age, sex, and race/ethnicity

Model 3: Adjusted for variables in the second model and low income, area-level income, smoking, diabetes, hypertension, chronic kidney disease, history of heart failure, dementia, depression, insomnia, cancer, epilepsy, and hospitalization within the past year

Model 4: Adjusted for variables in Model 3 and use of antihypertensive medication, diabetes medication, barbiturates, benzodiazepines, antihistamine medication for insomnia, non-benzodiazepine medication for insomnia, sedative hypnotics, and sedative antidepressants, statins, non-statin lipid-lowering therapy, and hormone replacement therapy

Opioids (47%) and antihypertensive medications (54%) were the most commonly used acute and preventive migraine medications among patients with a history of migraine, respectively (Supplemental Table 9). Also, 15% of patients with a history of migraine were taking a triptan. After multivariable adjustment and compared to patients without a history of migraine, patients with a history of migraine who were taking an NSAID, opioid medication, antiepileptic agent, antihypertensive medication, antidepressant and two or more migraine agents had a higher risk for ischemic stroke. Patients with a history of migraine taking a triptan had a lower risk for CHD and CVD events versus those without a history of migraine.

## Discussion

In the current analysis, the risk for ischemic stroke was higher among older adults with versus without a history of migraine. This association was present for patients with and without a history of CVD. Compared to patients without a history of migraine, the higher risk for ischemic stroke was present among patients with a history of migraine taking an opioid medication, but not among those taking a triptan. There was no evidence of a difference in the risk for CHD events for older adults with and without a history of migraine. However, patients with a history of migraine taking a triptan had lower risk for CHD events when compared to patients without migraine.

In previous studies of primarily middle-aged adults, migraine had been associated with an increased risk for stroke and CHD [1, 20–22]. In a meta-analysis of 16 cohort studies including more than one million participants, a history of migraine was associated with an increased risk for stroke (adjusted HR 1.41 [95% CI, 1.25–1.61]) and myocardial infarction (adjusted HR 1.23 [95% CI, 1.03–1.43]) [1]. However, substantial heterogeneity was present when comparing results across studies in this meta-analysis [1]. Few data are available examining the association of migraine with ischemic stroke and CHD events among older adults [1, 6]. In a study including 1919 adults ≥65 years of age, late-life non-migraine headache was associated with an increased risk for stroke [23]. There was no association between migraine and risk for stroke in this study. However, only 73 total stroke events, including two among those with migraine, occurred during follow up of this cohort. In another study that included 1292 adults (mean age 68 ± 9 years), migraine was associated with increased risk of stroke among smokers (adjusted HR 3.17; 95% CI 1.13, 8.85) but not in nonsmokers (adjusted HR 0.77; 95% CI 0.44, 1.35) [24]. In the current analysis, with a larger sample of older US adults with and without a history of migraine as well as thousands of stroke and CHD events that occurred during follow-up, a history of migraine was associated with a higher risk for ischemic stroke, but not with CHD events.

In a prior analysis of the Women’s Health Study, women who had migraine with aura had a higher risk for ischemic stroke compared to women without migraine [18]. However, women who had migraine without aura did not have an increased risk for ischemic stroke compared to those without migraine. The incidence rate of ischemic stroke in the current study was higher for individuals with a history of migraine, with and without aura, compared to those without a history of migraine. However, the current results need to be interpreted with caution as some patients with migraine with aura may have been misclassified as without aura if they did not have a specific diagnosis code for this condition.

Triptans are contraindicated for patients with a history of coronary artery disease, cerebrovascular disease or peripheral vascular disease, or with uncontrolled hypertension [8]. Consistent with prior studies, the use of triptans was lower among patients with a history of migraine included in the current analysis who had versus those who did not have a history of CVD [25, 26]. The use of triptans by patients with migraine was associated with a lower risk for CHD events compared to those without a history of migraine. This finding may be explained by physicians selectively prescribing triptans to those patients with a history of migraine perceived to be at a lower risk for future CVD events.

The use of opioids is not recommended by any migraine treatment guideline [27–30]. However, prior studies have shown that the use of opioids is high among middle-aged adults with migraine [31, 32]. In the current study, opioids were the acute migraine medications most commonly used by older adults with a history of migraine, both among those without and with a history of CVD. The use of opioids has been associated with an increased risk of CVD events [12–14]. This association may be mediated by opioid receptors in the cardiovascular system [33]. For example, it has been suggested that chronic use of opioid may increase the risk for ischemic stroke through an altered expression of tight junction proteins which could result in a disruption in the blood brain barrier [34]. In a study of Medicare beneficiaries from New Jersey and Pennsylvania, opioid use was associated with more than twice the risk of myocardial infarction compared with the use of NSAIDs [13]. Among patients with a history of CVD in the current study, those with history of migraine taking opioids had a higher risk of ischemic stroke and CHD events versus their counterparts without a history of migraine. However, we cannot rule out confounding by indication. This would occur, for example, if patients at high risk for CHD events were more likely to be treated with opioids versus other migraine medications.

In addition to the efficacy of a therapy, comorbid and coexisting conditions, potential adverse events, and patient preferences should be taken into consideration when developing a treatment plan for individuals with a history of migraine [28]. For many older individuals with a history of migraine, cardiovascular contraindications to migraine-specific therapies can pose substantial treatment challenges. Current treatment guidelines for episodic migraine do not provide specific recommendations for the management of older adults or those with increased CVD risk [27–30]. More specific recommendations for the treatment of older adults with migraine, particularly those with a history of CVD or high CVD risk, may lead to better migraine management by reducing the frequency, duration, and severity of migraine attacks and subsequently improving quality of life in this population.

The current study has several strengths. We included a large sample of adults ≥65 years with and without migraine. Since most older US adults have health insurance through Medicare, the current findings have a high degree of generalizability to older adults in the US [35]. Additionally, data on stroke, CHD, and CVD outcomes were available over 10 years of follow-up. We used a number of strategies to control for possible confounding and bias. We used the same inclusion/exclusion criteria and algorithms to identify outcome events among patients with and without migraine. We also used a matched design to control for differences in age, sex, and history of CVD. Lastly, we included numerous covariates in the statistical models to control potential residual confounding. However, the current study has several limitations. Patients with a history of migraine were identified using diagnosis codes and pharmacy claims data which may not capture individuals who are undiagnosed or who do not seek medical care for migraine. The current results may have been attenuated if patients with migraine were misclassified as not having migraine and still had CVD events. We were unable to assess time since migraine diagnosis, and attack frequency and severity for patient’s migraine attacks because these metrics are unavailable in claims data. We were unable to evaluate non-prescription treatment for alleviating migraine pain including over the counter medications, massage, hot or cold compresses, and nutraceuticals. Pharmacy claims can only identify that beneficiaries filled a prescription, but not that they actually took the medication. Additionally, many of these medications are taken as needed and we do not know how frequently patients used them. Finally, the preventive migraine medications and some acute migraine medications (e.g., opioids) included in this study have indications for other diseases. It is possible that patients with a history of migraine were taking these medications to treat other conditions.

## Conclusion

In the current study of Medicare beneficiaries, rates of ischemic stroke were higher among those with versus without migraine, but migraine was not associated with a higher risk for CHD events. The risk for ischemic stroke among patients with migraine differed by migraine medication classes. The current findings have implications for clinicians treating older individuals with migraine. These results suggest the need for more specific clinical guidelines and recommendations to appropriately treat older adults with migraine.

## Supplementary Information

**Additional file 1: Table S1.** Definition of a history of migraine. **Table S2.** Definition of patient characteristics. **Table S3.** Antihypertensive medications, glucose lowering medications, statins, non-statin lipid-lowering medication, medications for insomnia and hormone replacement therapy. **Table S4.** Migraine medications. **Table S5.** Definitions of ischemic stroke and coronary heart disease events. **Table S6.** Incidence rates and hazard ratios for ischemic stroke associated with a history of migraine with and without aura among patients without a history of cardiovascular disease. **Table S7.** Incidence rates and hazard ratios for ischemic stroke, coronary heart disease and cardiovascular disease associated with a history of migraine and migraine medication drug classes among patients without a history of cardiovascular disease. **Table S8.** Incidence rates and hazard ratios for risk of ischemic stroke associated with a history of migraine without and with aura among patients with a history of cardiovascular disease. **Table S9.** Incidence rates and hazard ratios for ischemic stroke, coronary heart disease and cardiovascular disease associated with a history of migraine and migraine medications among patients with a history of cardiovascular disease.

## Data Availability

The datasets generated and/or analyzed during the current study will be made available from the corresponding author upon reasonable request.

## Abbreviations

CHD: Coronary heart disease
CI: Confidence intervals
CKD: Chronic kidney disease
CMS: Centers for Medicare and Medicaid Services
CVD: Cardiovascular disease
HR: Hazard ratios
NSAID: Nonsteroidal anti-inflammatory drug
