# Supplementary material for: Risk for ischemic stroke and coronary heart disease associated with migraine and migraine medication among older adults
Source: J Headache Pain. 2021 Oct 13;22(1):124. doi: 10.1186/s10194-021-01338-z (PMC8513203; doi:10.1186/s10194-021-01338-z)
Supplement: Supplementary file 1 — Additional file 1: Table S1. Definition of a history of migraine. Table S2. Definition of patient characteristics. Table S3. Antihypertensive medications, glucose lowering medications, statins, non-statin lipid-lowering medication, medications for insomnia and hormone replacement therapy. Table S4. Migraine medications. Table S5. Definitions of ischemic stroke and coronary heart disease events. Table S6. Incidence rates and hazard ratios for ischemic stroke associated with a history of migraine with and without aura among patients without a history of cardiovascular disease. Table S7. Incidence rates and hazard ratios for ischemic stroke, coronary heart disease and cardiovascular disease associated with a history of migraine and migraine medication drug classes among patients without a history of cardiovascular disease. Table S8. Incidence rates and hazard ratios for risk of ischemic stroke associated with a history of migraine without and with aura among patients with a history of cardiovascular disease. Table S9. Incidence rates and hazard ratios for ischemic stroke, coronary heart disease and cardiovascular disease associated with a history of migraine and migraine medications among patients with a history of cardiovascular disease. [file 10194_2021_1338_MOESM1_ESM.docx]

**Supplemental Material**

Risk for ischemic stroke and coronary heart disease associated with migraine and migraine medication among older adults

Supplemental Table 1: Definition of a history of migraine.

| History of migraine was defined by meeting at least one of the following components between January 1, 2008 and December 31, 2017: | |
| --- | --- |
| - Had ≥1 inpatient claim with a diagnosis of migraine (ICD-9 diagnosis code of 346.xx, or ICD-10 diagnosis code of G43.xxx) in any discharge diagnosis position. | |
| - Had ≥1 outpatient evaluation and management claim with a diagnosis of migraine and a specialty code of 13 (neurologist). | |
| - Had ≥1 claim for emergency room visit with a diagnosis of migraine. | |
| - Had ≥1 outpatient evaluation and management claim with a diagnosis of migraine with ≥1 pharmacy fill for a migraine-specific triptan or an ergotamine class medication within 180 days of the date of diagnosis. A full list of migraine-specific triptans and ergotamine class medications is provided below. | |
| - Had ≥2 outpatient evaluation and management claims with a diagnosis of migraine between 7 and 180 days apart | |
| - Had ≥2 pharmacy fills for migraine-specific triptans or ergotamine class medications between 7 and 180 days apart | |
| List of migraine medications: | |
| **Drug category** | **Generic name** |
| **Acute migraine medications** | |
| Ergotamine class medications | Dihydroergotamine mesylate, Ergotamine tartrate, Ergotamine tartrate + caffeine, Ergotamine tartrate + caffeine + belladonna + pentobarbital |
| Migraine-specific triptans | Almotriptan, Eletriptan, Frovatriptan, Naratriptan, Rizatriptan, Sumatriptan, Zolmitriptan |

ICD-9: International Classification of Diseases, Ninth Revision; ICD-10: International Classification of Diseases, Tenth Revision.

Supplemental Table 2: Definition of patient characteristics.

| Variable | Definition |
| --- | --- |
| Age | Calculated on the index date using birth date from the Medicare beneficiary summary file. |
| Sex | As defined in the Medicare beneficiary summary file. |
| History of cardiovascular disease | Defined by a history of coronary heart disease or ischemic stroke as defined below:   1. History of coronary heart disease:   Any of the following using all available claims prior to each patient’s index date:   - 1. At least 1 inpatient claim with an ICD-9 diagnosis code of 410.xx-414.xx, V45.81 or V45.82, or an ICD-10 diagnosis code of I21.xxx, I22.xxx, I25.10, I25.810, I25.811, I25.812, I25.3, I25.41, I25.42, Z95.1 or Z9861 in any position.   2. At least 1 outpatient physician evaluation and management claim with an ICD-9 diagnosis code of 410.xx-414.xx, V45.81 or V45.82, or an ICD-10 diagnosis code of I21.xxx, I22.xxx, I25.10, I25.810, I25.811, I25.812, I25.3, I25.41, I25.42, Z95.1 or Z9861 in any position.   3. At least 1 inpatient or outpatient claim with an ICD-9 procedure code of 00.66, 36.0, 36.01-36.19, 36.2 or a CPT code of 33510-33519, 33521-33523, 33530, 33533-33536, 92980-92982, 92984, 92995, 92996, 92920, 92921, 92924, 92925, 92928, 92929, 92933, 92934, 92937, 92938, 92941, 92943, 92944, 92973, C9600, C9601, C9602, C9603, C9604, C9605, C9606, C9607, C9608  1. History of ischemic stroke:   Any one of the following using claims using all available claims prior to each patient’s index date:   - 1. ≥1 overnight inpatient claim with an ICD-9 discharge diagnosis code of 433.x1 or 434.x1, or an ICD-10 discharge diagnosis code of I63.xx in any discharge diagnosis position.   2. ≥2 outpatient claims with an ICD-9 diagnosis code of 433.x1 or 434.x1, or an ICD-10 diagnosis code of I63.xx in any position. |
| Race/ethnicity | As defined in the Medicare beneficiary summary file. |
| Low income subsidy | Receipt of any low-income subsidy under Medicare pharmacy program or a state reported dual eligible status code of 01-08 or a state buy-in code (value of ‘C’) from the entitlement variable for any month within 365 days prior to each patient’s index date. These codes refer to Medicare beneficiaries for whom Medicaid pays premiums. |
| Area-level income | Area-level income: Median income by zip code were obtained from 2017 American Community Survey data and merged to the Medicare claims using each beneficiary’s residential zip code. Area-level income were grouped into four categories: <$25,000, $25,000 - $49,999, $50,000 - 74,999, and ≥$75,000. |
| Smoking | Any of the following within 365 days prior to each patient’s index date.   1. ≥1 hospitalization with a discharge diagnosis code of tobacco use (ICD-9 diagnosis code of 305.1, 649.0x, 989.84, or V15.82, or ICD-10 CM diagnosis code of F17.200, F17.201, F17.210, F17.211, F17.220, F17.221, F17.290, F17.291, or Z87.891) in any discharge position. 2. ≥1 physician evaluation and management visit with a diagnosis code of tobacco use (ICD-9 diagnosis code of 305.1, 649.0x, 989.84, or V15.82, or ICD-10 diagnosis code of F17.200, F17.201, F17.210, F17.211, F17.220, F17.221, F17.290, F17.291, or Z87.891) in any position. 3. ≥1 hospitalization with a discharge diagnosis code or physician evaluation and management visit of tobacco use with a CPT code of 99406, 99407, G0436, G0437, G9016, S9453, S4995, G9276, G9458, 1034F, 4004F, 4001F. 4. ≥1 pharmacy claim for nicotine or varenicline. |
| Diabetes | Any of the following using all available claims prior to each patient’s index date:   1. At least 1 inpatient claim with a discharge ICD-9 diagnosis (any position) of 250.xx, 357.2, 362.0x, or 366.41 or an ICD-10 diagnosis (any position) of 'E0836', 'E0842', 'E0936', 'E0942', 'E1010', 'E1011', 'E1029', 'E10311', 'E10319', 'E1036', 'E1039', 'E1040', 'E1042', 'E1051', 'E10618', 'E10620', 'E10621', 'E10622', 'E10628', 'E10630', 'E10638', 'E10641', 'E10649', 'E1065', 'E1069', 'E108', 'E109', 'E1100', 'E1101', 'E1129', 'E11311', 'E11319', 'E11329', 'E11339', 'E11349', 'E11359', 'E1136', 'E1139', 'E1140', 'E1142', 'E1151', 'E11618', 'E11620', 'E11621', 'E11622', 'E11628', 'E11630', 'E11638', 'E11641', 'E11649', 'E1165', 'E1169', 'E118', 'E119', 'E1310', 'E1336', 'E1342'. 2. At least 2 carrier claims, carrier line or outpatient claims with ICD-9 diagnosis (any position) of 250.xx, 357.2, 362.0x, or 366.41 or ICD-10 diagnosis (any position) of 'E0836', 'E0842', 'E0936', 'E0942', 'E1010', 'E1011', 'E1029', 'E10311', 'E10319', 'E1036', 'E1039', 'E1040', 'E1042', 'E1051', 'E10618', 'E10620', 'E10621', 'E10622', 'E10628', 'E10630', 'E10638', 'E10641', 'E10649', 'E1065', 'E1069', 'E108', 'E109', 'E1100', 'E1101', 'E1129', 'E11311', 'E11319', 'E11329', 'E11339', 'E11349', 'E11359', 'E1136', 'E1139', 'E1140', 'E1142', 'E1151', 'E11618', 'E11620', 'E11621', 'E11622', 'E11628', 'E11630', 'E11638', 'E11641', 'E11649', 'E1165', 'E1169', 'E118', 'E119', 'E1310', 'E1336', 'E1342', linked by CLAIM_ID to an ambulatory physician evaluation and management claim, with the 2 claims occurring at least 7 days apart. 3. At least 1 pharmacy claim for an oral antidiabetic drug fill or insulin (see Supplemental Table 2). |
| Hypertension | Any of the following using all available claims before each patient’s index date:   1. ≥1 inpatient claim with an ICD-9 discharge diagnosis code of 401.x, 403.0x, 403.1x, 403.9x or an ICD-10 discharge diagnosis code of I10, I12.0, I12.9 in any discharge diagnosis position. 2. ≥2 outpatient claims with an ICD-9 diagnosis code of 401.x, 403.0x, 403.1x, 403.9x or an ICD-10 diagnosis code of I10, I12.0, I12.9 in any position at least 30 days apart. |
| Chronic kidney disease | Any of the following using all available claims prior to each patient’s index date (this definition is from the United States Renal Data System [USRDS] annual report):   1. ≥1 inpatient claim with a discharge diagnosis code of chronic kidney disease (ICD-9 diagnosis code of 016.0, 095.4, 189.0, 189.9, 223.0, 236.91, 250.4, 271.4, 274.1, 283.11, 403.x1, 403.x0, 404.x2, 404.x3, 404.x0, 404.x1, 440.1, 442.1, 447.3, 572.4, 580–588, 591, 642.1, 646.2, 753.12–753.17, 753.19, 753.2, 794.4 or ICD-10 diagnosis code of ‘A1811', 'A5275', 'C649', 'C689', 'D3000', 'D4100', 'D4120', 'D593', 'E1021’, 'E1029', 'E1121’, 'E1129', 'E748', 'I120', 'I129', 'I130', 'I1310', 'I1311', 'I132', I701', 'I722', 'K767', 'M1030', 'N003', 'N008', 'N009', 'N013', 'N022', 'N032', 'N033', 'N035', 'N038', 'N039', 'N040', 'N043', 'N044', 'N048', 'N049', 'N052', 'N055', 'N058', 'N059', 'N08', 'N1330', 'N170', 'N171', 'N172', 'N178', 'N179', 'N181', 'N182', 'N183', 'N184', 'N185', 'N186', 'N189', 'N19', 'N250', 'N251', 'N2581', 'N2589', 'N259', 'N269','Q6102', 'Q6119', 'Q612', 'Q613', 'Q614', 'Q615', 'Q618', 'Q6210', 'Q6211', 'Q6212', 'Q6231', 'Q6239', 'R944') in any discharge diagnosis position. 2. ≥1 physician evaluation and management visit with a diagnosis code of chronic kidney disease (ICD-9 diagnosis code of 016.0, 095.4, 189.0, 189.9, 223.0, 236.91, 250.4, 271.4, 274.1, 283.11, 403.x1, 403.x0, 404.x2, 404.x3, 404.x0, 404.x1, 440.1, 442.1, 447.3, 572.4, 580–588, 591, 642.1, 646.2, 753.12–753.17, 753.19, 753.2, or 794.4 or ICD-10 diagnosis code of ‘A1811', 'A5275', 'C649', 'C689', 'D3000', 'D4100', 'D4120', 'D593', 'E1021’, 'E1029', 'E1121’, 'E1129', 'E748', 'I120', 'I129', 'I130', 'I1310', 'I1311', 'I132', I701', 'I722', 'K767', 'M1030', 'N003', 'N008', 'N009', 'N013', 'N022', 'N032', 'N033', 'N035', 'N038', 'N039', 'N040', 'N043', 'N044', 'N048', 'N049', 'N052', 'N055', 'N058', 'N059', 'N08', 'N1330', 'N170', 'N171', 'N172', 'N178', 'N179', 'N181', 'N182', 'N183', 'N184', 'N185', 'N186', 'N189', 'N19', 'N250', 'N251', 'N2581', 'N2589', 'N259', 'N269','Q6102', 'Q6119', 'Q612', 'Q613', 'Q614', 'Q615', 'Q618', 'Q6210', 'Q6211', 'Q6212', 'Q6231', 'Q6239', 'R944') in any position. 3. Additionally, if the flag ESRD_IND in the Master beneficiary summary file is checked then the participant will be categorized as having a history of CKD. |
| History of heart failure | Any of the following using all available claims prior to each patient’s index date:   1. ≥ 1 inpatient claim with ICD-9 diagnoses (any position) of 402.01, 402.11, 402.91, 404.01, 404.03, 404.11, 404.13, 404.91, 404.93, 428.X, or ICD-10 diagnoses (any position) of 'I110', 'I130', 'I132', 'I501', 'I5020', 'I5021', 'I5022', 'I5023', 'I5030', 'I5031', 'I5032', 'I5033', 'I5040', 'I5041', 'I5042', 'I5043', 'I509', or 2. ≥ 2 outpatient or carrier claims on separate calendar days with ICD-9 diagnoses (any position) of 402.01, 402.11, 402.91, 404.01, 404.03, 404.11, 404.13, 404.91, 404.93, 428.X, or ICD-10 diagnoses (any position) of 'I110', 'I130', 'I132', 'I501', 'I5020', 'I5021', 'I5022', 'I5023', 'I5030', 'I5031', 'I5032', 'I5033', 'I5040', 'I5041', 'I5042', 'I5043', 'I509' |
| Depression | Any of the following using claims within 365 days prior to each patient’s index date:   1. At least 1 inpatient claim with ICD-9 diagnosis code (any position) of 296.20-296.26, 296.30-296.36, 296.51-296.56, 296.60-296.66, 296.89, 298.0, 300.4, 309.1 or 311 or ICD-10 diagnosis code (any position) of F32.9, F32.0-F32.5, F33.9, F33.0-F33.3, F33.41, F33.42, F33.31, F31.32, F31.4, F31.5, F31.75, F31.76, F31.60-F31.64, F31.77, F31.78, F31.81, F32.3 or F33.3, F34.1, F43.21 or F32.9. 2. At least 1 E/M outpatient claim with ICD-9 diagnosis code (any position) of 296.20-296.26, 296.30-296.36, 296.51-296.56, 296.60-296.66, 296.89, 298.0, 300.4, 309.1 or 311, or ICD-10 diagnosis code (any position) of F32.0-F32.5, F33.9, F33.0-F33.3, F33.41, F33.42, F33.31, F31.32, F31.4, F31.5, F31.75, F31.76, F31.60-F31.64, F31.77, F31.78, F31.81, F32.3 or F33.3, F34.1, F43.21 or F32.9. |
| Anxiety disorders | Defined by at least 1 inpatient claim or 1 outpatient emergency and evaluation claim with ICD-9 diagnosis code 293.84, 300.00, 300.01, 300.02, 300.09, 300.10, 300.20, 300.21, 300.22, 300.23, 300.29, 300.3, 300.5, 300.89, 300.9, 308.0, 308.1, 308.2, 308.3, 308.4, 308.9, 309.81, 313.0, 313.1, 313.21, 313.22, 313.3, 313.82, 313.83 or ICD-10 diagnosis code of F064, F4000, F4001, F4002, F4010, F4011, F40210, F40218, F40220, F40228, F40230, F40231, F40232, F40233, F40240, F40241, F40242, F40243, F40248, F40290, F40291, F40298, F408, F409, F410, F411, F413, F418, F419, F42, F422, F423, F424, F428, F429, F430, F4310, F4311, F4312, F488, F489, R452, R453, R454, R455, R456, R457, R4581, R4582, R4583, R4584 within 365 days prior to each patient’s index date. |
| Insomnia | Defined by at least one inpatient or outpatient claim with ICD-9 diagnosis code of 307.41, 307.42, 307.49, 327.00, 327.01, 327.09, 780.52, or V69.4 or ICD-10 diagnosis code of F51.02 or F51.09, F51.01, F51.8, G47.01, G47.09, or Z72.820 within 365 days prior to each patient’s index date. |
| History of dementia | Any of the following using all available claims prior to each patient’s index date:   1. ≥1 hospitalization with a discharge diagnosis code of dementia (ICD-9 diagnosis code of 331.0, 331.1, 331.2, 331.7, 290.0, 290.1, 290.11, 290.12, 290.13, 290.20, 290.21, 290.3, 290.40, 290.41, 290.42, 290.43, 294.0, 294.1, 294.8, or 797 or ICD-10 diagnosis code of 'F0150', 'F0151', 'F0280', 'F0281', 'F0390', 'F0391', ‘G300', 'G301', 'G308', 'G309', 'G3101', 'G3109', ‘G311’, F04, F068, G331, G94) in any discharge diagnosis position. 2. ≥1 physician evaluation and management visit with a diagnosis code of dementia (ICD-9 diagnosis code of 331.0, 331.1, 331.2, 331.7, 290.0, 290.1, 290.11, 290.12, 290.13, 290.20, 290.21, 290.3, 290.40, 290.41, 290.42, 290.43, 294.0, 294.1, 294.8, or 797 or ICD-10 diagnosis code of 'F0150', 'F0151', 'F0280', 'F0281', 'F0390', 'F0391', ‘G300', 'G301', 'G308', 'G309', 'G3101', 'G3109', ‘G311’, F04, F068, G331, G94) in any position. |
| Cancer | Any of the following using claims within 365 days prior to each patient’s index date:   1. ≥1 inpatient or outpatient emergency room ICD-9 diagnosis code of 140.xx – 172.xx, 174.xx – 208.xx, 209.0 – 209.3 or ICD10 diagnosis code of C00.xx-C96.xx, or D03.xx in any position. 2. ≥2 outpatient ICD9- diagnosis codes (outpatient or carrier files) with same first 3 digits in (140.xx – 172.xx, 174.xx – 208.xx, 209.0 – 209.3) or ICD10 diagnosis code of C00.xx-C96.xx, or D03.xx in any position associated with a physician evaluation and management code 30 - 365 days apart 3. Treatment with chemotherapy or radiation therapy: any single inpatient or outpatient claim with ICD-9, ICD-10, HCPCS or CPT codes for chemotherapy, radiation, or hormone therapy. 4. ≥1 Inpatient, outpatient emergency room or carrier/outpatient claim associated with physician Evaluation & Management/Health Care Common Procedure Coding System code for ICD-9 diagnosis code of V10.xx (history of malignant neoplasm) or ICD-10 diagnosis code of Z85.xx or Z85.xxx (history of malignant neoplasm). |
| Epilepsy | Any of the following using all available claims prior to each patient’s index date:   1. ≥1 inpatient, outpatient or carrier claim with an ICD-9 diagnosis code of 345.xx or ICD-10 diagnosis code of G40.xx in any diagnosis position. Outpatient and carrier claims should be linked to an evaluation and management code. 2. ≥2 inpatient, outpatient or carrier claims with an ICD-9 diagnosis code of 780.3x or ICD-10 diagnosis code of R56.xx in any diagnosis position on different dates. Outpatient and carrier claims should be linked to an evaluation and management code. |
| Hospitalization in the prior year | Defined by ≥1 inpatient claim within 365 days prior to each patient’s index date. |
| History of migraine with aura | 1. Had ≥1 inpatient claim with a diagnosis of migraine with aura (ICD-9 diagnosis code of 346.0x, 346.5x and 346.6x or ICD-10 diagnosis code of G43.1xx, G43.5xx and G43.6xx). 2. Had ≥1 outpatient evaluation and management claim with a diagnosis of migraine with aura and a specialty code of 13 (neurologist). 3. Had ≥1 claim for emergency room visit with a diagnosis of migraine with aura. 4. Had ≥2 outpatient evaluation and management claims with a diagnosis of migraine with aura between 7 and 180 days apart. |

ICD-9: International Classification of Diseases, Ninth Revision; ICD-10: International Classification of Diseases, Tenth Revision; CPT: Current Procedural Terminology.

Supplemental Table 3: Antihypertensive medications, glucose lowering medications, statins, non-statin lipid-lowering medication, medications for insomnia and hormone replacement therapy.

| **Drug category** | **Generic name** |
| --- | --- |
| **Antihypertensive medications** |  |
| Thiazide or thiazide-type diuretics | Chlorothiazide, Chlorthalidone, Hydrochlorothiazide, Indapamide, Metolazone,  Hydrochlorothiazide (HCTZ) |
| ACE inhibitors | Benazepril, Captopril, Enalapril, Fosinopril, Lisinopril, Moexipril, Perindopril, Quinapril, Ramipril, Trandolapril |
| ARBs | Azilsartan, Candesartan, Eprosartan, Irbesartan, Losartan, Olmesartan,  Telmisartan, Valsartan |
| Calcium channel blockers | Amlodipine, Felodipine, Isradipine, Nicardipine, Nifedipine, Nisoldipine, Diltiazem, Verapamil |
| Diuretics | Bumetanide, Furosemide, Torsemide, Amiloride, Triamterene, Eplerenone,  Spironolactone, Aliskiren |
| Beta blockers | Atenolol, Betaxolol, Bisoprolol, Metoprolol, Nebivolol, Nadolol, Propanolol,  Acebutolol, Carteolol, Penbutolol, Pindolol, Carvedilol, Labetalol |
| Alpha-1 blockers | Doxazosin, Prazosin, Terazosin |
| Direct vasodilators | Hydralazine, Minoxidil |
| **Glucose-lowering medications** | Acarbose, Acetohexamide, Albiglutide, Alogliptin, Canagliflozin, Chlorpropamide, Dapagliflozin, Dulaglutide, Empagliflozin, Ertugliflozin,  Exenatide, Exenatide ER, Glibenclamide, Glimepiride, Glipizide, Glyburide,  Inhaled insulin, Insulin, Insulin Aspart, Insulin Degludec, Insulin Detemir, Insulin Glargine, Insulin Glulisine, Insulin Human NPH, Insulin Human Regular, Insulin Lispro, Linagliptin, Liraglutide, Lixisenatide, Metformin, Miglitol, Nateglinide,  Pioglitazone, Pramlintide, Repaglinide, Rosiglitazone, Saxagliptin, Semaglutide,  Sitagliptin, Tolazamide, Tolbutamide, Cycloset (brand name for bromocriptine). |
| **Statins** | Atorvastatin, Fluvastatin, Lovastatin, Pravastatin, Rosuvastatin, Simvastatin |
| **Non-statin lipid-lowering medications** | Ezetimibe, Fibrates, Niacin, or Bile acid sequestrants |
| **Medications for Insomnia** |  |
| Barbiturates | Amobarbital, Butabarbital, Pentobarbital, Secobarbital, Phenobarbital, Mephobarbital |
| Benzodiazepines | Alprazolam, Clonazepam, Estazolam, Flurazepam, Lorazepam, Oxazepam, Quazepam, Temazepam, Triazolam |
| Antihistamine | Hydroxyzine, Diphenhydramine |
| Z drugs | Eszopiclone, Zaleplon, Zolpidem |
| Sedative hypnotics | Chloral hydrate |
| Sedative antidepressants | Amitriptyline, Nortriptyline, Doxepin, Clomipramine, Trazodone, Nefazodone,  Mirtazapine |
| **Hormone replacement therapy (among women)** | Estradiol, Progesterone, Medroxyprogesterone, Estropipate, Drosperinone, Etonorgestrel, Levonorgestrel, Norethindrone, Conjugated Estrogens/ Medroxyprogesterone Acetate, Esterified Estrogens And Methyltestosterone, Conjugated estrogens, Conjugated Estrogens Synthetic A, Conjugated Estrogens Synthetic B, Conjugated Estrogens/Bazedoxifene, Esterified Estrogens |

ACE: angiotensin-converting-enzyme; ARB: angiotensin II receptor blockers.

Supplemental Table 4: Migraine medications.

| **Drug category** | **Generic name** |
| --- | --- |
| **Acute migraine medications** | |
| Ergotamine class medications | Dihydroergotamine mesylate, Ergotamine tartrate, Ergotamine tartrate + caffeine, Ergotamine tartrate + caffeine + belladonna + pentobarbital |
| Migraine-specific triptans | Almotriptan, Eletriptan, Frovatriptan, Naratriptan, Rizatriptan, Sumatriptan, Zolmitriptan |
| NSAIDs^*^ | Celecoxib, Diclofenac, Diflunisal, Etodolac, Fenoprofen, Flurbiprofen, Ibuprofen, Indomethacin, Ketoprofen, Ketorolac, Meclofenamic acid, Mefenamic acid, Meloxicam, Nabumetone, Naproxen, Oxaprozin, Piroxicam, Rofecoxib, Salsalate, Sulindac, Tolmetin, Valdecoxib |
| Opioids^†^ | Codeine, Fentanyl, Hydrocodone, Hydromorphone, Morphine sulfate, Oxycodone, Oxymorphone, Tramadol, Alfentanil, Buprenorphine, Butorphanol tartrate, Dezocine, Dihydrocodeine, Levomethadyl, Levorphanol, Meperidine, Methadone, Nalbuphine, Opium / belladonna alkaloids / opium alkaloids, Pentazocine, Propoxyphene, Remifentanil, Sufentanil, Tapentadol |
| **Preventive migraine medications** | |
| Anti-epileptic agents | Carbamazepine, Gabapentin, Levetiracetam, Pregabalin, Topiramate, Valproate sodium / valproic acid / divalproex sodium, Zonisamide |
| Antihypertensive agents^‡^ | Atenolol, Bisoprolol, Metoprolol, Nadolol, Nebivolol, Pindolol, Propranolol, Timolol, Verapamil, Candesartan, Clonidine, Lisinopril |
| Antidepressants | Duloxetine, Desvenlafaxine, Venlafaxine, Amitriptyline, Desipramine, Doxepin, Imipramine, Nortriptyline, Protriptyline, Escitalopram, Citalopram, Sertraline |
| Botulinum toxin^§^ | AbobotulinumtoxinA, IncobotulinumtoxinA, OnabotulinumtoxinA, RimabotulinumtoxinB |
| Other migraine preventive agents^‡^ | Carisoprodol, Cyproheptadine, Guanfacine, Memantine, Methysergide, Milnacipran, Tizanidine |

NSAID: nonsteroidal anti-inflammatory drugs.

^*^ Main NSAID generic names are listed for brevity. The analysis included drugs that contain the generic drug plus other ingredients.

^†^ Main opioid generic names are listed for brevity. The analysis included drugs that contain the generic drug plus other ingredients as well as various formulations.

^‡^ Flunarizine was not analyzed as this medication is not available in the US. Erenumab, fremanezumab, galcanezumab, and eptinezumab were not analyzed as they were not commercially available during the study period.

^§^ In addition to pharmacy fills for botulinum toxin medications, we also included any outpatient or carrier claim with a Current Procedural Terminology code of 64615

Supplemental Table 5: Definitions of ischemic stroke and coronary heart disease events.

| Event | Definition |
| --- | --- |
| Ischemic stroke | Defined by an inpatient claim with a discharge diagnosis code for ischemic stroke (i.e., ICD-9 codes 433.x1 and 434.x1 or ICD-10 codes of I63.xx) as recorded in the primary discharge diagnosis position. |
| Coronary heart disease event | Defined by a myocardial infarction hospitalization or a coronary revascularization.  Myocardial infarction hospitalization is defined by an overnight hospitalization with a discharge diagnosis code for myocardial infarction (i.e., an ICD-9 code 410.xx, except 410.x2, which represent a subsequent episode of care, or an ICD-10 of code I21.xx, or I22.xx) in any discharge diagnosis position.  Coronary revascularization is defined by:   1. An inpatient or outpatient claim with an ICD-9 procedure code of 00.66, 36.0, 36.01-36.19, 36.2, an ICD-10 procedure code starting with any of the following 4 digits: 0210, 0211, 0212, 0213, 0270, 0271, 0272, 0273, 02C0, 02C1, 02C2, 02C3, 3E07, or 2. A CPT code for coronary revascularization. CPT codes for coronary revascularization change by calendar year. CPT codes for coronary revascularization in any calendar year include 33510-33519, 33521-33523, 33530, 33533-33536. CPT codes for coronary revascularization in or before 2012 include 92980-92982, 92984, 92995, and 92996. CPT codes for coronary revascularization in or after 2013 include 92920, 92921, 92924, 92925, 92928, 92929, 92933, 92934, 92937, 92938, 92941, 92943, and 92944. |

ICD-9: International Classification of Diseases, Ninth Revision; ICD-10: International Classification of Diseases, Tenth Revision; CPT: Current Procedure Terminology

Supplemental Table 6: Incidence rates and hazard ratios for ischemic stroke associated with a history of migraine with and without aura among patients without a history of cardiovascular disease.

|  | History of migraine | | |
| --- | --- | --- | --- |
|  | No  (n=87,960) | Yes, without aura  (n=19,333) | Yes, with aura  (n = 2,657) |
| Ischemic stroke |  |  |  |
| Number of events | 1383 | 358 | 48 |
| Follow-up in person-years | 342,211 | 76,459 | 10,267 |
| Incidence rate (95% CI)^*^ | 4.04 (3.83, 4.25) | 4.68 (4.20, 5.17) | 4.68 (3.35, 6.00) |
| Hazard ratio (95% CI) |  |  |  |
| Model 1 | 1 (ref) | 1.16 (1.03, 1.30) | 1.15 (0.86, 1.54) |
| Model 2 | 1 (ref) | 1.22 (1.09, 1.37) | 1.14 (0.86, 1.53) |
| Model 3 | 1 (ref) | 1.22 (1.08, 1.37) | 1.17 (0.88, 1.57) |
| Model 4 | 1 (ref) | 1.21 (1.07, 1.37) | 1.18 (0.88, 1.57) |

* Rates are expressed per 1,000 person-years. Model 1: Unadjusted model.

Model 2: Adjusted for age, race/ethnicity, and sex.

Model 3: Adjusted for variables in the second model and low income, area-level income, tobacco use, diabetes, hypertension, CKD, history of heart failure, dementia, depression, insomnia, cancer, epilepsy, and hospitalization within the past year.

Model 4: Adjusted for variables in Model 3 and use of antihypertensive medication, diabetes medication, barbiturates, benzodiazepines, antihistamine medication for insomnia, non-benzodiazepine medication for insomnia, sedative hypnotics, and sedative antidepressants, statins, non-statin lipid-lowering therapy, and hormone replacement therapy.

Supplemental Table 7. Incidence rates and hazard ratios for ischemic stroke, coronary heart disease and cardiovascular disease associated with a history of migraine and migraine medication drug classes among patients without a history of cardiovascular disease.

|  | | Incidence rate per 1,000 person-years (95% CI) | | |
| --- | --- | --- | --- | --- |
|  | n (%) | Ischemic stroke | CHD^*^ | Composite CVD events |
| No history of migraine | 87,960 (100%) | 4.0 (3.8, 4.3) | 8.4 (8.1, 8.7) | 12.1 (11.7, 12.4) |
| History of migraine | 21,990 (100%) |  |  |  |
| Not taking migraine medication | 3,097 (14%) | 4.9 (3.7, 6.2) | 7.6 (6.1, 9.2) | 12.5 (10.5, 14.5) |
| Taking migraine medication ^†^ |  |  |  |  |
| Triptan | 7,905 (36%) | 2.5 (2.0, 3.0) | 5.4 (4.6, 6.2) | 7.7 (6.7, 8.6) |
| NSAID | 4,268 (19%) | 4.4 (3.4, 5.4) | 8.4 (7.0, 9.7) | 12.4 (10.7, 14.1) |
| Opioid | 7,867 (36%) | 5.7 (4.8, 6.5) | 10.4 (9.3, 11.5) | 15.7 (14.3, 17.1) |
| Migraine-preventive antiepileptic agents | 4,698 (21%) | 4.6 (3.6, 5.6) | 10.2 (8.7, 11.7) | 14.6 (12.8, 16.5) |
| Migraine-preventive antihypertensive agents | 8,079 (37%) | 5.8 (4.9, 6.6) | 9.8 (8.7, 10.9) | 15.0 (13.7, 16.4) |
| Migraine-preventive antidepressants | 6,394 (29%) | 4.4 (3.6, 5.3) | 8.4 (7.3, 9.6) | 12.5 (11.1, 13.9) |
| Other migraine-preventive agents | 1,160 (5%) | 5.0 (2.9, 7.1) | 10.0 (7.0, 12.9) | 14.3 (10.7, 17.8) |
| ≥ 2 agents | 12,234 (56%) | 4.5 (3.9, 5.1) | 8.6 (7.8, 9.4) | 12.7 (11.7, 13.7) |
|  | | Multivariable-adjusted hazard ratios (95% CI) | | |
| No history of migraine | 87,960 (100%) | 1 (ref) | 1 (ref) | 1 (ref) |
| History of migraine | 21,990 (100%) |  |  |  |
| Not taking migraine medication | 3,097 (14%) | 1.29 (0.99, 1.67) | 0.95 (0.77, 1.18) | 1.09 (0.93, 1.29) |
| Taking migraine medication ^†^ |  |  |  |  |
| Triptan | 7,905 (36%) | 0.86 (0.68, 1.08) | 0.79 (0.67, 0.93) | 0.82 (0.71, 0.93) |
| NSAID | 4,268 (19%) | 1.21 (0.95, 1.53) | 1.00 (0.85, 1.19) | 1.07 (0.93, 1.23) |
| Opioid | 7,867 (36%) | 1.43 (1.20, 1.69) | 1.13 (1.00, 1.28) | 1.22 (1.10, 1.35) |
| Migraine-preventive antiepileptic agents | 4,698 (21%) | 1.18 (0.93, 1.50) | 1.12 (0.96, 1.32) | 1.16 (1.02, 1.33) |
| Migraine-preventive antihypertensive agents | 8,079 (37%) | 1.21 (1.03, 1.43) | 1.01 (0.89, 1.14) | 1.06 (0.96, 1.17) |
| Migraine-preventive antidepressants | 6,394 (29%) | 1.19 (0.96, 1.48) | 1.00 (0.85, 1.16) | 1.06 (0.93, 1.20) |
| Other migraine-preventive agents | 1,160 (5%) | 1.13 (0.74, 1.74) | 1.04 (0.77, 1.42) | 1.04 (0.80, 1.35) |
| ≥ 2 agents | 12,234 (56%) | 1.16 (0.99, 1.35) | 1.00 (0.89, 1.11) | 1.04 (0.95, 1.14) |

CI: Confidence Interval; CHD: coronary heart disease; CVD: cardiovascular disease; NSAID: Nonsteroidal anti-inflammatory drug.

* Includes myocardial infarction or coronary revascularization.

† Few patients with migraine were taking ergotamine (n=128) and botulinum toxin (n=196) to analyze these groups separately.

Multivariable models adjust for: age, sex, race/ethnicity, low income, area-level income, smoking, diabetes, hypertension, CKD, history of heart failure, dementia, depression, insomnia, cancer, epilepsy, hospitalization within the past year, use of antihypertensive medication, diabetes medication, barbiturates, benzodiazepines, antihistamine medication for insomnia, non-benzodiazepine medication for insomnia, sedative hypnotics, and sedative antidepressants, statins, non-statin lipid-lowering therapy, and hormone replacement therapy

Supplemental Table 8: Incidence rates and hazard ratios for risk of ischemic stroke associated with a history of migraine without and with aura among patients with a history of cardiovascular disease.

|  | History of migraine | | |
| --- | --- | --- | --- |
|  | No  (n=63,612) | Yes, without aura  (n=13,658) | Yes, with aura  (n=2,245) |
|  |  |  |  |
| Number of events | 2,195 | 655 | 110 |
| Follow-up in person-years | 227,257 | 49,524 | 8,573 |
| Incidence rate (95% CI) ^*^ | 9.66 (9.25, 10.06) | 13.23 (12.21, 14.24) | 12.83 (10.43, 15.23) |
| Hazard ratio (95% CI) |  |  |  |
| Model 1 | 1 (ref) | 1.37 (1.26, 1.50) | 1.34 (1.10, 1.62) |
| Model 2 | 1 (ref) | 1.40 (1.28, 1.52) | 1.35 (1.11, 1.64) |
| Model 3 | 1 (ref) | 1.25 (1.14, 1.37) | 1.26 (1.04, 1.53) |
| Model 4 | 1 (ref) | 1.27 (1.16, 1.39) | 1.29 (1.06, 1.57) |

^*^ Rates are expressed per 1,000 person-years. Model 1: Unadjusted model.

Model 2: Adjusted for age, race/ethnicity, and sex.

Model 3: Adjusted for variables in the second model and low income, area-level income, tobacco use, diabetes, hypertension, CKD, history of heart failure, dementia, depression, insomnia, cancer, epilepsy, and hospitalization within the past year.

Model 4: Adjusted for variables in Model 3 and use of antihypertensive medication, diabetes medication, barbiturates, benzodiazepines, antihistamine medication for insomnia, non-benzodiazepine medication for insomnia, sedative hypnotics, and sedative antidepressants, statins, non-statin lipid-lowering therapy, and hormone replacement therapy.

Supplemental Table 9: Incidence rates and hazard ratios for ischemic stroke, coronary heart disease and cardiovascular disease associated with a history of migraine and migraine medications among patients with a history of cardiovascular disease.

|  | | Incidence rate per 1,000 person-years (95% CI) | | |
| --- | --- | --- | --- | --- |
|  | n (%) | Ischemic stroke | CHD^*^ | Composite CVD events |
| No history of migraine | 63,612 (100%) | 9.7 (9.3, 10.1) | 29.0 (28.3, 29.8) | 37.8 (36.9, 38.6) |
| History of migraine | 15,903 (100%) |  |  |  |
| Not taking migraine medication | 1,884 (12%) | 11.9 (9.3, 14.5) | 23.3 (19.6, 26.9) | 34.4 (29.9, 39.0) |
| Taking migraine medication ^†^ |  |  |  |  |
| Triptan | 2,350 (15%) | 7.8 (6.0, 9.6) | 22.9 (19.8, 25.9) | 29.7 (26.2, 33.3) |
| NSAID | 3,045 (19%) | 11.8 (9.9, 13.8) | 26.3 (23.4, 29.3) | 37.7 (34.1, 41.2) |
| Opioid | 7,479 (47%) | 13.0 (11.7, 14.4) | 33.5 (31.3, 35.8) | 45.7 (43.0, 48.3) |
| Migraine-preventive antiepileptic agents | 4,626 (29%) | 13.2 (11.4, 15.1) | 34.0 (31.0, 37.0) | 46.1 (42.6, 49.6) |
| Migraine-preventive antihypertensive agents | 8,527 (54%) | 14.4 (13.1, 15.8) | 34.3 (32.2, 36.4) | 47.7 (45.2, 50.2) |
| Migraine-preventive antidepressants | 5,195 (33%) | 13.8 (12.1, 15.5) | 30.7 (28.2, 33.3) | 43.7 (40.6, 46.8) |
| Other migraine-preventive agents | 1,108 (7%) | 13.3 (9.6, 17.0) | 32.6 (26.7, 38.4) | 45.8 (38.7, 52.9) |
| ≥ 2 agents | 9,832 (62%) | 13.0 (11.8, 14.2) | 32.2 (30.3, 34.1) | 44.2 (42.0, 46.5) |
|  | | Multivariable-adjusted hazard ratios (95% CI) | | |
| No history of migraine | 63,612 (100%) | 1 (ref) | 1 (ref) | 1 (ref) |
| History of migraine | 15,903 (100%) |  |  |  |
| Not taking migraine medication | 1,884 (12%) | 1.29 (1.03, 1.61) | 0.92 (0.79, 1.09) | 1.02 (0.89, 1.17) |
| Taking migraine medication ^†^ |  |  |  |  |
| Triptan | 2,350 (15%) | 0.93 (0.74, 1.18) | 0.83 (0.72, 0.95) | 0.85 (0.75, 0.96) |
| NSAID | 3,045 (19%) | 1.20 (1.01, 1.43) | 0.90 (0.80, 1.01) | 0.99 (0.89, 1.09) |
| Opioid | 7,479 (47%) | 1.21 (1.07, 1.36) | 1.01 (0.93, 1.08) | 1.06 (0.99, 1.13) |
| Migraine-preventive antiepileptic agents | 4,626 (29%) | 1.17 (1.01, 1.36) | 1.01 (0.92, 1.11) | 1.05 (0.97, 1.14) |
| Migraine-preventive antihypertensive agents | 8,527 (54%) | 1.28 (1.15, 1.42) | 1.01 (0.95, 1.09) | 1.08 (1.02, 1.15) |
| Migraine-preventive antidepressants | 5,195 (33%) | 1.34 (1.17, 1.54) | 0.96 (0.87, 1.05) | 1.05 (0.97, 1.14) |
| Other migraine-preventive agents | 1,108 (7%) | 1.20 (0.90, 1.60) | 1.04 (0.86, 1.25) | 1.10 (0.94, 1.29) |
| ≥2 agents | 9,832 (62%) | 1.21 (1.09, 1.35) | 0.99 (0.93, 1.06) | 1.05 (0.99, 1.11) |

CI: Confidence Interval; CHD: coronary heart disease; CVD: cardiovascular disease; NSAID: Nonsteroidal anti-inflammatory drug.

* Includes myocardial infarction or coronary revascularization.

† Few patients with migraine were taking ergotamine (n=54) and botulinum toxin (n=85). Therefore, these groups were not analyzed separately.

Multivariable models adjust for: age, sex, race/ethnicity, low income, area-level income, smoking, diabetes, hypertension, CKD, history of heart failure, dementia, depression, insomnia, cancer, epilepsy, hospitalization within the past year, use of antihypertensive medication, diabetes medication, barbiturates, benzodiazepines, antihistamine medication for insomnia, non-benzodiazepine medication for insomnia, sedative hypnotics, and sedative antidepressants, statins, non-statin lipid-lowering therapy, and hormone replacement therapy
